# *Strongyloides stercoralis*: Spatial distribution of a highly prevalent and ubiquitous soil-transmitted helminth in Cambodia

**DOI:** 10.1371/journal.pntd.0006943

**Published:** 2019-06-20

**Authors:** Armelle Forrer, Virak Khieu, Penelope Vounatsou, Paiboon Sithithaworn, Sirowan Ruantip, Rekol Huy, Sinuon Muth, Peter Odermatt

**Affiliations:** 1 Swiss Tropical and Public Health Institute, Basel, Switzerland; 2 University of Basel, Basel, Switzerland; 3 National Centre for Parasitology, Entomology and Malaria Control, Ministry of Health, Phnom Penh, Cambodia; 4 Department of Parasitology and Cholangiocarcinoma Research Institute, Faculty of Medicine, Khon Kaen University, Khon Kaen, Thailand; 5 Graduated School of Biomedical Science, Khon Kaen University, Khon Kaen, Thailand; Istituto Superiore di Sanità, ITALY

## Abstract

**Background:**

*Strongyloides stercoralis* is a neglected soil-transmitted helminth that occurs worldwide, though it is particularly endemic in tropical and subtropical areas. It can cause long-lasting and potentially fatal infections due to its ability to replicate within its host. *S*. *stercoralis* causes gastrointestinal and dermatological morbidity. The objective of this study was to assess the *S*. *stercoralis* infection risk and, using geostatistical models, to predict its geographical distribution in Cambodia.

**Methodology / Principal findings:**

A nation-wide, community-based parasitological survey was conducted among the Cambodian population, aged 6 years and older. *S*. *stercoralis* was diagnosed using a serological diagnostic test that detects IgG antibodies in urine. Data on demography, hygiene and knowledge about helminth infection were collected. *S*. *stercoralis* prevalence among 7,246 participants with a complete data record was 30.5%, ranging from 10.9% to 48.2% across provinces. The parasite was ubiquitous in Cambodia; only five south-eastern provinces had prevalence rates below 20%. Infection risk increased with age for both men and women, although girls under the age of 13 and women aged 50 years and over had lower odds of infection than their male counterparts. Open defecation was associated with higher odds of infection, while having some knowledge of the health problems caused by worms was a protective factor. Infection risk was positively associated with nighttime maximum temperature, minimum rainfall, and distance to water; it was negatively associated with land occupied by rice fields.

**Conclusions / Significance:**

*S*. *stercoralis* infection is rampant in Cambodia. Control programs delivering ivermectin are needed to manage the parasite. However, the high cost of this drug in Cambodia currently precludes the implementation of control initiatives. Donations, subsidies or affordable generics are needed so that *S*. *stercoralis*, which infects almost a third of the Cambodian population, can be addressed through an adequate control program.

## Author summary

The threadworm, *Strongyloides stercoralis*, is a highly neglected worm infection, transmitted through infective larvae in the soil. Threadworms occur worldwide, particularly in tropical climates. It may cause long-lasting and potentially fatal infections due to the parasite’s ability to replicate within its host. This study aimed to assess the risk of threadworm infection at national level in Cambodia. We conducted a nation-wide, community-based parasitological survey of the Cambodian population, aged 6 years and over. The threadworm was diagnosed using a serological diagnostic test that detects antibodies in urine. Data on demography, hygiene and knowledge about helminth infection were collected. The purpose of this study was to predict the risk of *S*. *stercoralis* infection in unsurveyed locations, assess risk factors for infection, and map its geographical distribution in Cambodia. About one third (30.5%) of the enrolled study participants (n = 7,246) were infected with threadworms. At provincial level, the lowest and highest infection rates were 10.9% and 48.2%, respectively. Prevalence rates below 20% were found in just five south-eastern provinces. The risk of a threadworm infection increased with age for both men and women. Open defecation was associated with higher risk of infection, while having some knowledge of the health problems caused by worms was a protective factor. Infection risk was positively associated with environmental factors, such as nighttime maximum temperature, minimum rainfall, and distance to water; it was negatively associated with land occupied by rice fields. Threadworm infection is highly prevalent in Cambodia and adequate control measures, including access to treatment, are warranted to address the burden of this Neglected Tropical Disease (NTD) in Cambodia.

## Introduction

*Strongyloides stercoralis* is a highly neglected intestinal nematode, for which larvae living in soil polluted with feces infect humans transcutaneously, like hookworms. *S*. *stercoralis* occurs worldwide but thrives in warm regions with poor sanitation conditions and has been under-detected and overlooked for decades because its larvae are not uncovered by standard field diagnostic techniques [1–5]. Until recently, the only available prevalence estimates originated from a review conducted in the late 80s, which estimated some 30–100 million cases worldwide [6]. More recent estimates show prevalence rates between 10% and 40% in subtropical and tropical countries [1]. Using diagnostic approaches suitable for detecting *S*. *stercoralis*, some studies indicate that the prevalence of *S*. *stercoralis* could be half that of hookworm, i.e. 200–370 million cases worldwide [1, 7, 8].

In Cambodia, two community-based, large-scale surveys documented *S*. *stercoralis* prevalence rates of 25% and 45% in the southern province of Takeo and in the northern province of Preah Vihear, respectively [9, 10]. *S*. *stercoralis* infection is more prevalent among adults due to its unique ability among soil-transmitted helminths (STHs) to replicate within the host, which leads to infections that can last for decades in the absence of treatment [11]. In cases of immunosuppression, this auto-infection cycle accelerates and results in hyperinfection, a condition that is 100% fatal if left untreated [12–14]. Chronic infection with *S*. *stercoralis* may cause abdominal pain, nausea, vomiting, and diarrhea, as well as urticaria and larva currens [15–17]. The latter is a serpiginous intermittent moving eruption due to parasite migration under the skin. Its location on the buttocks, thighs, and trunk, together with the high speed of migration (i.e. 5 to 10 centimeters an hour), makes it a symptom highly specific to strongyloidiasis [11, 13]. Finally, although this aspect of infection needs to be confirmed, *S*. *stercoralis* infection might be associated with growth retardation in children [17]. Due to this combination of significant morbidity and high prevalence, *S*. *stercoralis* has been recognized as a public health problem in Cambodia. However, the national prevalence and the location of high-risk zones are unknown.

One of the most sensitive diagnostic approaches combines the Baermann and Koga agar plate culture techniques, but this method is costly, time consuming and requires laboratory staff specifically trained to identify *S*. *stercoralis* larvae by microscopy [10, 18, 19]. Serological diagnosis is more sensitive than most coprological approaches, but its use may be limited in endemic settings due to cross-reactions with other helminths species [20, 21]. Likewise, serology may overestimate prevalence in endemic areas as it detects parasite-specific antibodies that remain long after contact with the parasite or cure, and cannot distinguish current from past infections [20]. While this last aspect would be an issue for cure assessment, it does not affect prevalence estimates in a population naïve to treatment against the investigated parasite. A serological test was recently developed in Thailand, using an antigen from *S*. *ratti* to detect antibodies in urine [22, 23]. This technique has several strengths. First, collecting urine samples is much easier than collecting fecal samples. Second, this test has a high sensitivity of 93% when compared with coprological methods. Lastly, there is little cross-reactivity with other STH species or food-borne trematodes, including *Opisthorchis viverrini* [22, 23]. However, like other serological tests, it does not differentiate between active and past infections.

In the past decade, geostatistical models have increasingly been used to delineate risk zones for helminthic infections, at small and large scale, and to help target control efforts in areas with the highest need [24–31]. Based on the association between environmental variables and infection levels at survey locations, such models can be used to predict infection levels throughout entire geographical zones.

A national parasitological survey was conducted in 2016, in all provinces of Cambodia, to assess *S*. *stercoralis* prevalence based on a serological diagnostic test using *S*. *ratti* antigens [22]. Using these data, this work set out to estimate *S*. *stercoralis* prevalence in Cambodia, to assess risk factors for infection, and to predict *S*. *stercoralis* infection risk throughout the country to help guide control efforts.

## Methods

### Ethics statement

The study was approved by the National Ethics Committee for Health Research, Ministry of Health, Cambodia (NECHR, reference number 188, dated 02.05.2016). Prior to enrolment, all participants received an explanation of the study goals and procedures. All participants aged 16 years and over provided written informed consent, while parents or legal guardians provided consent for participants aged 6–15 years. All *S*. *stercoralis* cases were treated with a single oral dose of ivermectin (200μg/kg BW) and all other diagnosed parasitic infections were treated according to the national guidelines [32].

### Study setting

Cambodia counted 15.6 million inhabitants in 2015, 79.3% of whom lived in rural areas [33]. The country has undergone rapid economic development in recent decades. With a Human Development Index ranking of 143/188 in 2016, Cambodia belonged to the group of lower middle-income countries, as per the World Bank classification [33, 34]. Poverty levels have decreased dramatically in recent years, with the proportion of the population living in extreme poverty falling to 2.2% in 2016. However, approximately one person in five (21.6%) lives on less than USD 3.1/day [33]. Adult literacy and net enrolment in primary school were 74% and 95%, respectively, in 2010–2014, while 32% of children under the age of 59 months were stunted in 2015 [34]. In 2015, 42% and 69% of the rural population had access to improved sanitation facilities and improved water sources, respectively, while those figures were 88% and 100% for the urban population, respectively [34].

### Study population and design

A cross-sectional, community-based survey was conducted among the general population in all 25 provinces of Cambodia, between May and August 2016. In each province, 10 villages were selected from all villages using a simple random sampling procedure in STATA version 13.0 (StataCorp LP; College Station, United States of America). In each village selected, seven to eight households were randomly selected based on the list of households obtained from the village chief. Eighteen villages were originally selected and subsequently replaced because their remote locations compromised the quality of collected samples for parasitological data. In each village, households were selected using systematic proportional sampling; all household members present on the survey day were enrolled up to a maximum of 35 participants per village. All household members aged 6 years and over were eligible.

### Assessment of *Strongyloides stercoralis* infection

Participants were asked to provide a urine sample, from which *S*. *stercoralis* was diagnosed using an enzyme-linked immunosorbent assay (ELISA) based on *S*. *ratti* antigens [22]. After collection, urine specimens were preserved in NaN^3^ with a final concentration of 0.1%, and kept at 4°C until required for analysis. Samples were sent to the central laboratory of the National Centre for Parasitology, Entomology and Malaria Control (CNM) in Phnom Penh and then to Khon Kaen University, Thailand, for ELISA testing.

### Individual risk factor data

An individual questionnaire was administered to all study participants and covered demographics (age, sex, level of education, main occupation), the number of household members, access to sanitation (latrine availability at home, usual place of defecation) and knowledge of worm infections (transmission route of and health problems caused by helminths) (S1 Appendix, English version of questionnaire).

### Environmental data

Environmental parameters were extracted from freely available remote sensing (RS) sources for the period September 2015 to August 2016, corresponding to one year prior to the last month of the study. Daytime and nighttime land surface temperature (LST), international geosphere biosphere programme (IGBP) type 1 land use/land cover (LULC), as well as normalized difference vegetation index (NDVI), and enhanced vegetation index (EVI) were extracted at 1 x 1 km resolution from Moderate Resolution Imaging Spectroradiometer (MODIS) Land Processes Distributed Active Archive Center (LP DAAC), U.S. Geological Survey (USGS) Earth Resources Observation and Science (EROS) Center (http://lpdaac.usgs.gov). Rainfall data was obtained from WorldClim (www.worldclim.org). Digital elevation data were retrieved from the NASA Shuttle Radar Topographic Mission (SRTM) and CGIAR-CSI database. Distance to large bodies of water was obtained from Health Mapper.

### Data management

Laboratory and questionnaire data were double-entered and validated in EpiData version 3.1 (EpiData Association; Odense, Denmark). Environmental data processing, geo-referencing and maps were done in ArcGIS version 10.2.1 (ESRI; Redlands, CA, United States). LULC 18 classes were merged into four categories, according to similarity and respective frequencies. Annual and seasonal means, as well as maxima and minima of monthly EVI, LST and RFE means were calculated and standardized. Environmental data were linked to parasitological and questionnaire data, according to geo-referenced locations. Data management and non-Bayesian data analysis were done in STATA version 13.0. Bayesian geostatistical models were fitted using WinBUGS version 1.4.3 (Imperial College & Medical Research Council; London, UK). Predictions for unsurveyed locations were performed in Fortran 95 (Compaq Visual Fortran Professional version 6.6.0, Compaq Computer Corporation; Houston, United States of America). Five age groups were established as follows: (i) 6–12 years, (ii) 13–18 years, (iii) 19–30 years, (iv) 31–50 years, and (v) >50 years.

### Statistical analysis

Chi-square (χ^2^) test was used to compare proportions. The association between infection risk and covariates was assessed using mixed non spatial bivariate logistic regressions, accounting for village clustering, i.e. with a non-spatial, village-level random effect. Covariates exhibiting an association at a significance level of at least 15%, as determined by the likelihood ratio test (LRT), were included in the multivariate logistic regression models. In the event of correlated variables, the variable resulting in the model with the smallest Akaike’s information criterion (AIC) was selected. For the risk factor analysis, variables exhibiting high Wald p-values were removed one by one and kept outside of the model if their removal resulted in a lower AIC. Summary measures of continuous environmental variables (i.e. LST day and night, rainfall, and distance to water) were standardized before inclusion in the multiple regression models. To explore the relationship between *S*. *stercoralis* infection risk and age, smoothed age-prevalence curves were produced with the “mkspline” command in STATA that regresses each outcome against a new age variable containing a restricted cubic spline of age.

For geostatistical models, a stationary isotropic process was assumed, with village-specific random effects following a normal distribution with mean zero, and a variance-covariance matrix that is an exponential function of the distance between pairs of locations. Vague prior distributions were chosen for all parameters. Further information on model specification is available in S2 Appendix. Markov chain Monte Carlo (MCMC) simulation was used to estimate model parameters [35]. Geostatistical models were run using the WinBUGS “spatial.unipred” function [36]. Convergence was assessed by examining the ergodic averages of selected parameters. For all models, a burn-in of 5,000 was followed by 30,000 iterations, after which convergence was reached. Results were withdrawn for the last 10,000 iterations of each chain, with a thinning of 10. Model fit was appraised with the Deviance Information Criterion (DIC). A lower DIC indicates a better model [37].

Three types of Bayesian mixed logistic models were run. First, models without covariates but using alternatively a geostatistical or an exchangeable random effect were run to quantify the extent of village-level spatial correlation and unexplained variance of *S*. *stercoralis* prevalence. Second, a risk factor analysis model was used to assess individual-level demographic, sanitation, and knowledge risk factors, as well as environmental covariates associated with infection risk. Third, a model including only environmental covariates was used to predict infection risk at unsurveyed locations.

### Prediction of *S*. *stercoralis* at unurveyed locations

To validate the model, 199 (80%) randomly selected villages were used for fitting, and the remaining 50 (20%) were used as test locations. A pair of models containing the same covariates, but including alternately a non-spatial (exchangeable) or spatial (geostatistical) random effect, was run. The predictive ability of the model was assessed by comparing the Mean Squared Error (MSE), which is obtained by squaring the average of absolute differences between predicted and observed prevalence rates at test locations.

Using the model with the best predictive ability, *S*. *stercoralis* infection risk was predicted at 68,410 pixels of 2x2 km resolution, using Bayesian Kriging [38].

## Results

### Study population

Among the 8,661 participants enrolled in the study, 1,407 did not provide any urine (one entire village was excluded due to all 34 of its participants not providing urine), 338 were removed because they did not provide a stool sample (requested for other assessments not presented in this work), and eight participants did not provide questionnaire data. Overall, 7,246 participants living in 2,585 households and 249 villages were included in the analysis. The mean number of participants per village was 30.2, with an interquartile range of six, and a minimum of five. With the exception of Ou Tracheak Chet in Preah Sihanouk Province (five participants) and Kampong Chrey in Preah Vihear province (nine participants), all villages had more than 10 participants and 93.6% of villages had 20 participants or more. Table 1 shows the characteristics of participants with complete parasitological and questionnaire data.

**Table 1:** Characteristics of the 7,246 participants included in the analysis.

Variable	Category	N (%)
Sex	Male	3,081 (42.5)
	Female	4,165 (57.5)
Age (years)	6–12	1,747 (24.1)
	13–18	954 (13.2)
	19–30	1,142 (15.8)
	31–50	1,850 (25.5)
	>50	1,553 (21.4)
Usual place of defecation	Toilet	4,961 (68.5)
	Forest	1,768 (24.4)
	River, rice field, other	517 (7.1)
Level of education	Primary school	4,183 (57.7)
	No school	1,279 (17.7)
	Secondary	1,283 (17.7)
	High school and beyond	501 (6.9)
Main occupation	Farmer	3,879 (53.5)
	At school	2,488 (34.4)
	At home	343 (4.7)
	Other	536 (7.4)
Any knowledge about worms	No	3,103 (42.8)
	Yes	4,143 (57.2)
Knowledge about sources of worm infection	No	3,995 (55.1)
	Yes	3,251 (44.9)
Knowledge about health problems caused by worms	No	4,507 (62.2)
	Yes	2,739 (37.8)
Knowledge about walking barefoot as a cause of worm infection	No	5,889 (81.3)
	Yes	1,357 (18.7)
Knowledge about lack of hygiene as a cause of infection	No	5,100 (70.4)
	Yes	2,146 (29.6)
Knowledge about open defecation as a cause of worm infection	No	5,993 (82.7)
	Yes	1,253 (17.3)
Knowledge about not washing hands as a cause of worm infection	No	5,796 (80.0)
	Yes	1,450 (20.0)
Toilet at home	No	2,306 (31.8)
	Yes	4,940 (68.2)

Data were obtained from a 2016 cross-sectional survey of individuals aged 6 years and older, living in 249 villages across the 25 provinces of Cambodia.

Females (57.5%) were overrepresented in the sample, compared to their proportion in the Cambodian population (51.5%) as assessed by the 2013 inter-census population survey [39]. The age distribution of the sample was very similar to that of the total Cambodian population: children and adolescents aged 14 years and younger represented 29.95% and 29.4% of the sample and of the Cambodian population, respectively; adolescents and adults aged 15 to 64 years represented 65.6% and 64.2%; and elderly adults aged 65 and older represented 5.8% and 5.0%.

The proportion of males and females were similar in the groups excluded from and included in the analysis; children and young adults aged 6–30 years were less represented (53.0%) in the sample than among the excluded participants (64.3%). Similarly, farmers were overrepresented (53.6% of the sample *vs*. 41.1% of excluded participants), while scholars were underrepresented (34.3% of the sample *vs*. 51.6% of excluded participants) in the final sample. In terms of usual place of defecation, there was no difference between participants excluded from or included in the analysis.

### *Strongyloides stercoralis* prevalence

**Fig 1:**
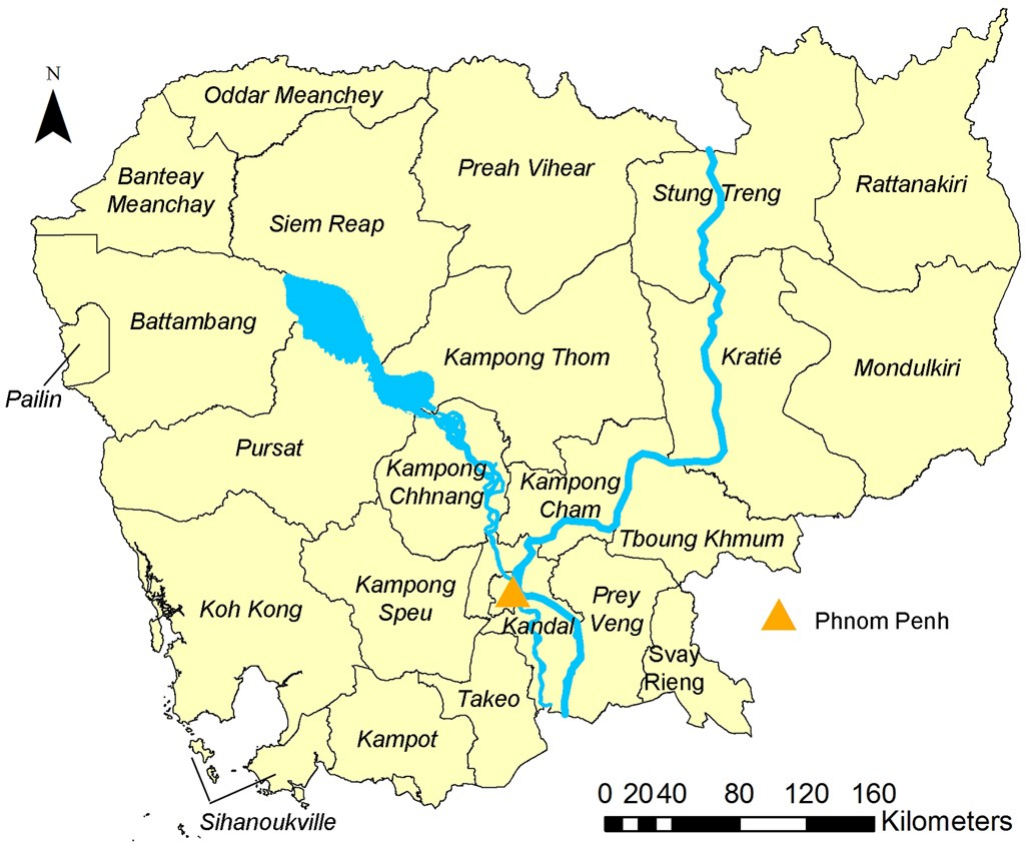
Map of Cambodian provinces. This map was created with ArcGIS version 10.0 (ESRI; Redlands, CA, USA) specifically for this study by Forrer et al.

**Fig 2:**
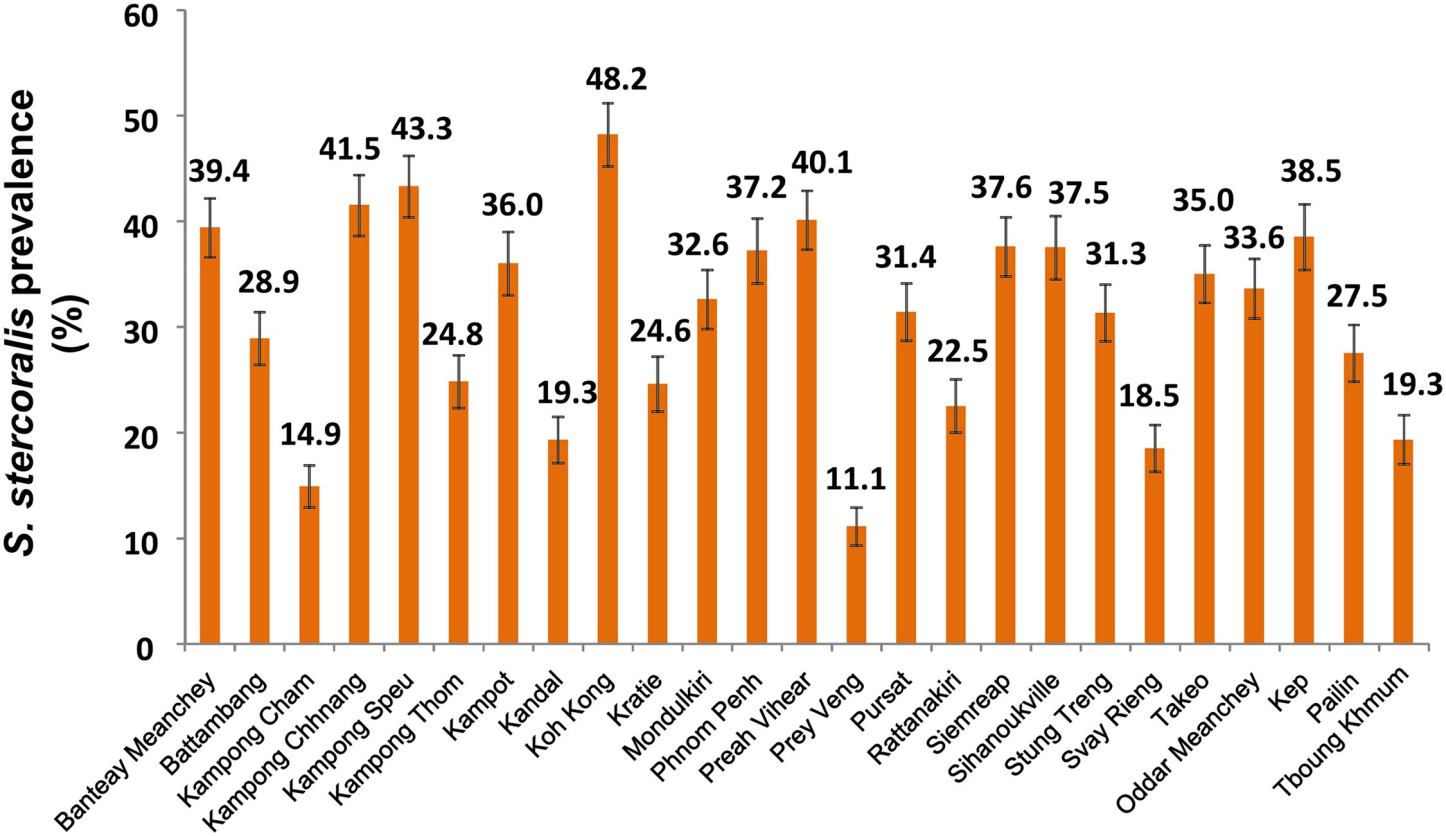
Provincial-level *S*. *stercoralis* prevalence in 25 provinces of Cambodia. Data were obtained from a 2016 cross-sectional survey of 7,246 participants aged 6 years and older, living in 249 villages across Cambodia.

**Fig 3:**
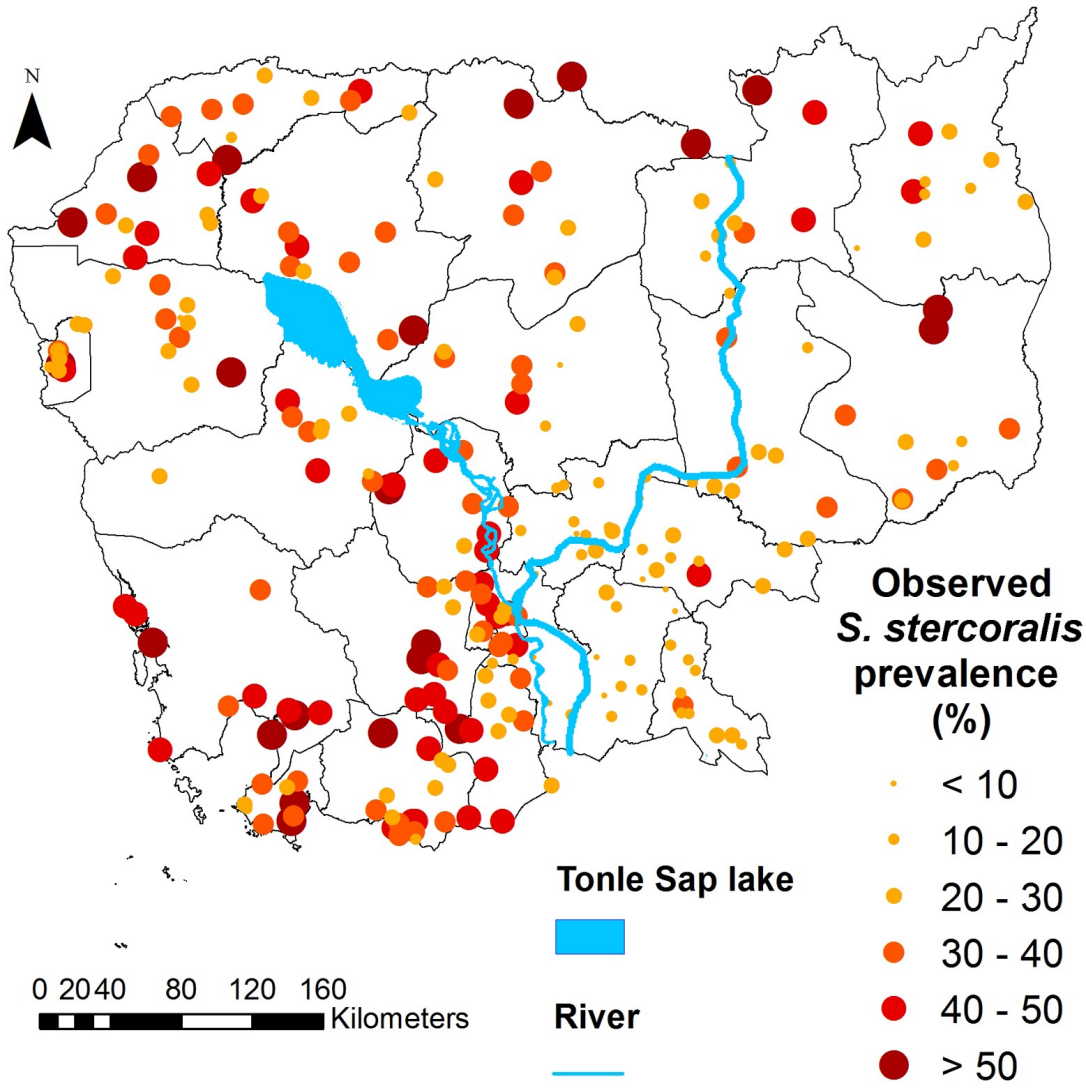
Map of Cambodia showing observed *S*. *stercoralis* prevalence in the 249 study villages. Data were obtained from a 2016 cross-sectional survey of 7,246 participants aged 6 years and older, living in 249 villages across Cambodia. This map was created with ArcGIS version 10.0 (ESRI; Redlands, CA, USA) and displays the results obtained specifically from this study by Forrer et al.

Overall, *S*. *stercoralis* prevalence was 30.7% (95% confidence interval (CI): 29.7–31.8), ranging from 10.9% (95%CI: 7.4–14–4) in Prey Veng province, to 48.2% (95%CI: 42.2–54.1) in Koh Kong province. Fig 1 shows the provinces of Cambodia and Fig 2 displays provincial level prevalence rates. Prevalence was highly variable at village level. The smallest prevalence rate of 2.9% (95% CI: 0.1–14.9) was found in a village in Kandal province, where only 1 of 35 participants was infected. The highest prevalence rates were 88.9% (95%CI: 51.8–99.7) and 80% (95%CI: 63.1–91.6), observed in villages in Preah Vihear and Koh Kong province, respectively. There were, however, only nine participants in the village in Preah Vihear province, resulting in a very large confidence interval. The map in Fig 3 shows the observed *S*. *stercoralis* prevalence in each village surveyed.

### Spatial correlation

Table 2 presents the model parameters of three geostatistical models, i.e. (i) model without covariates, (ii) the predictive model including only environmental variables, and (iii) the risk factor analysis model including environmental, demographic and behavioral covariates. In the absence of explanatory variables, *S*. *stercoralis* risk clustered at a distance of 85 km (range). The range dropped to 3.2 km after introducing environmental variables (predictive model).

**Table 2:** Parameters of three geostatistical models.

	No covariates ^(a)^		Predictive model ^(b)^		Risk factor analysis model ^(c)^	
	median	95% BCI	median	95% BCI	median	95% BCI
Range (km)	85.3	1.10–185.8	3.20	1.10–99.4	2.80	1.10–49.7
ρ	3.8	1.78–240.0	105.60	3.35–282.6	116.10	6.55–283.60
σ^2^	0.36	0.21–0.59	0.27	0.19–0.40	0.29	0.21–0.41

σ^2^ is the location-specific unexplained variance.

ρ is the decay parameter. The range (range = 3/ρ) is the distance at which the spatial correlation becomes less than 5%.

^(a)^ Geostatistical model without covariates

^(b)^ Predictive model: with environmental covariates only

^(c)^ Risk factor analysis model: with environmental and demographic and behavioral covariates

### Result of model validation and the predictive model

The predictive ability of the geostatistical model (MSE = 182.9, DIC = 6894.3) including environmental covariates (predictive model) was slightly higher than that of its non-spatial counterpart (MSE = 187.7, DIC = 6894.4). Therefore, the geostatistical model was used to predict *S*. *stercoralis* risk at unsurveyed locations. The geographical distributions of the covariates used in the geostatistical predictive model, together with elevation (which was not included in the predictive model), are shown in S1 Fig. The predictive model is presented in Table 3.

**Table 3:** Results of the geostatistical predictive model.

Variable	Category	OR	95% BCI
LST night dry season maximum (°C)	-	**1.21**	1.05–1.33
Rainfall year minimum (mm/month)	-	**1.35**	1.10–1.49
Distance to water (km)	-	1.10	0.97–1.22
Land use, land cover	Crops & natural vegetation mosaic, grass	1	-
	Cropland	0.82	0.67–1.03
	Forest and savanna	1.17	0.89–1.52
	Water and wetlands	1.29	0.88–1.91
Range (km)	-	3.20	1.10–99.4
ρ	-	105.6	3.35–282.6
σ^2^	-	0.27	0.19–0.40

LST: Land surface temperature; BCI, Bayesian credible interval; OR: odds ratio; OR in bold are significant at 5% level

σ^2^ is the location-specific unexplained variance.

ρ is the decay parameter. The range (range = 3/ρ) is the distance at which the spatial correlation becomes less than 5%.

### Risk factors for *S*. *stercoralis* infection

The results of the non-spatial bivariate mixed regressions are presented in S1 Table. Variables that were not significant in the multivariate model and whose removal decreased the model AIC were removed from the multivariate risk factor model during the model building process. The results of the multivariate Bayesian geostatistical risk factor analysis are presented in Table 4.

**Table 4:** Results of the risk factor analysis.

		*S*. *stercoralis* negative	*S*. *stercoralis* positive		
		N = 5,019	N = 2,227		
**Variable**	**Category**	**n (%)**	**n (%)**	**OR**	**95% BCI**
Sex ^(a)^	Male	2,138 (69.4)	943 (30.6)	1.00	-
	Female	2,881 (69.2)	1,284 (30.8)	0.87	0.67–1.12
Effect of age among men (years) ^(b)^	6–12	746 (82.8)	155 (17.2)	1.00	-
	13–18	335 (73.6)	120 (26.4)	**1.9**	1.44–2.50
	19–30	301 (72.0)	117 (28.0)	**2.14**	1.61–2.85
	31–50	452 (64.1)	253 (35.9)	**3.15**	2.49–4.07
	≥ 50	304 (50.5)	298 (49.5)	**6.11**	4.82–7.85
Interaction: effect of age among women (years)					
	6–12	717 (84.8)	129 (15.2)	1.00	-
	13–18	381 (76.4)	118 (23.6)	**1.89**	1.41–2.51
	19–30	505 (79.8)	219 (30.2)	**2.67**	2.06–3.46
	31–50	715 (62.5)	430 (37.5)	**4.01**	3.17–5.10
	≥ 50	563 (59.2)	388 (40.8)	**4.79**	3.77–6.07
Interaction: females compared to males, in each age group					
	6–12	-	-	1.00	-
	13–18	-	-	0.86	0.64–1.18
	19–30	-	-	1.08	0.82–1.43
	31–50	-	-	1.1	0.89–1.36
	≥ 50	-	-	**0.68**	0.55–0.85
Usual place of defecation	Toilet	3,503 (70.6)	1,458 (29.4)	1.00	-
	Forest	1,180 (66.7)	588 (33.3)	**1.24**	1.06–1.45
	River, rice field, other	336 (65.0)	181 (35.0)	**1.41**	1.12–1.80
Knowledge of signs of worm infection	No	3,117 (69.2)	1,390 (30.8)	1.00	-
	Yes			**0.86**	0.75–0.98
Land use, land cover	Crops & natural vegetation mosaic, grass	2,676 (69.0)	1,204 (31.0)	1.00	-
	Cropland	1,281 (73.8)	456 (26.2)	**0.81**	0.64–0.997
	Forest and savanna	802 (65.0)	431 (35.0)	1.21	0.92–1.57
	Water and wetlands	260 (65.7)	136 (34.3)	1.27	0.86–1.91
		**Median (IQR)**	**Median (IQR)**		
LST night dry season maximum (°C)	-	26.1 (1.4)	27.0 (1.6)	**1.22**	1.09–1.35
Rainfall year minimum (mm/month)	-	0.81 (0.70)	0.89 (0.91)	**1.38**	1.23–1.53
Distance to water (km)	-	14.9 (26.2)	16.0 (31.1)	**1.12**	1.01–1.25
**Model parameters**				**Median**	**95% BCI**
Range (km)	-	-	-	2.80	1.10–49.7
ρ	-	-	-	116.1	6.55–283.60
σ^2^	-	-	-	0.2914	0.21–0.41

LST: Land surface temperature; BCI, Bayesian credible interval; OR: odds ratio; OR in bold are significant at 5% level

σ^2^ is the location-specific unexplained variance.

ρ is the decay parameter. The range (range = 3/ρ) is the distance at which the spatial correlation becomes less than 5%.

^(a)^ Main effect of sex. Due to the interaction, the OR corresponds to the effect of sex among the baseline age group (6–12 years).

^(b)^ Main effect of age. Due to the interaction, the OR corresponds to the effect of age among males.

Results were obtained with the multivariate geostatistical model and data from a cross-sectional survey conducted in 2016 among 7,246 participants living in 249 villages across the 25 provinces of Cambodia.

**Fig 4:**
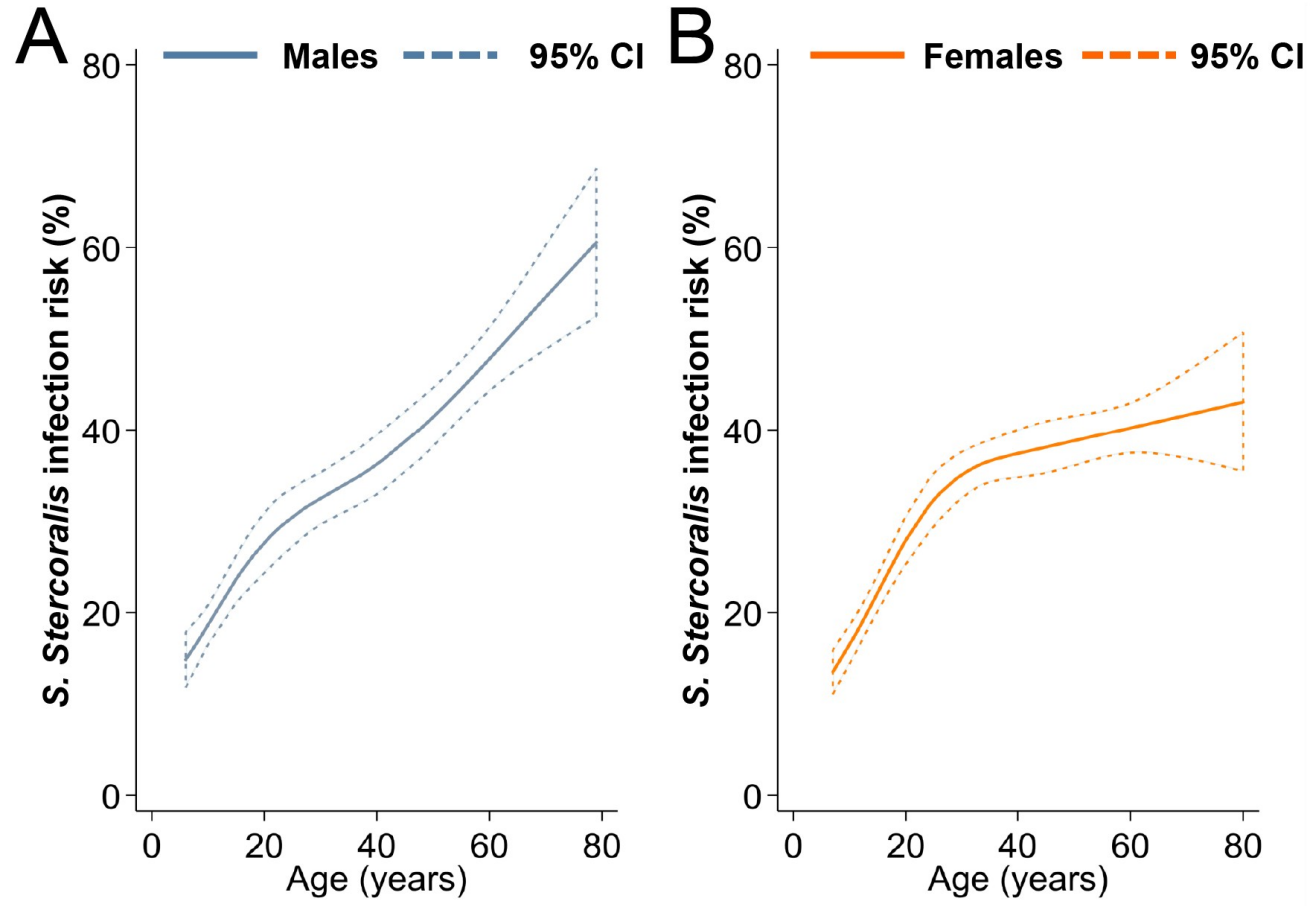
Smoothed age-prevalence of *S*. *stercoralis*, Cambodia. Data were obtained from a cross-sectional survey conducted in 2016 among 7,246 participants aged 6 years and older, living in 249 villages across Cambodia. Restricted cubic splines were used. Data are stratified for males (A) and females (B). Uncertainty is expressed as 95% confidence interval (CI).

Sex was an effect modifier of age. Infection risk increased with age for both sexes, but women aged 50 years and older had a lower risk of infection than males. The relationship between *S*. *stercoralis* infection risk and age is presented in Fig 4. Participants who practiced open defecation (31.5% of participants defecated either in forests, rice fields or water) had higher odds of infection, while individuals who had some knowledge about the health problems resulting from worm infection had lower odds of harboring *S*. *stercoralis*. Regarding environmental factors, *S*. *stercoralis* infection risk was positively associated with increasing nighttime land surface temperature (LST night) dry season maximum, increasing minimum annual rainfall, and increasing distance to water. Finally, the odds of *S*. *stercoralis* infection were lower among participants living in villages located in croplands (rice fields).

### Spatial prediction of *S*. *stercoralis* infection risk

**Fig 5:**
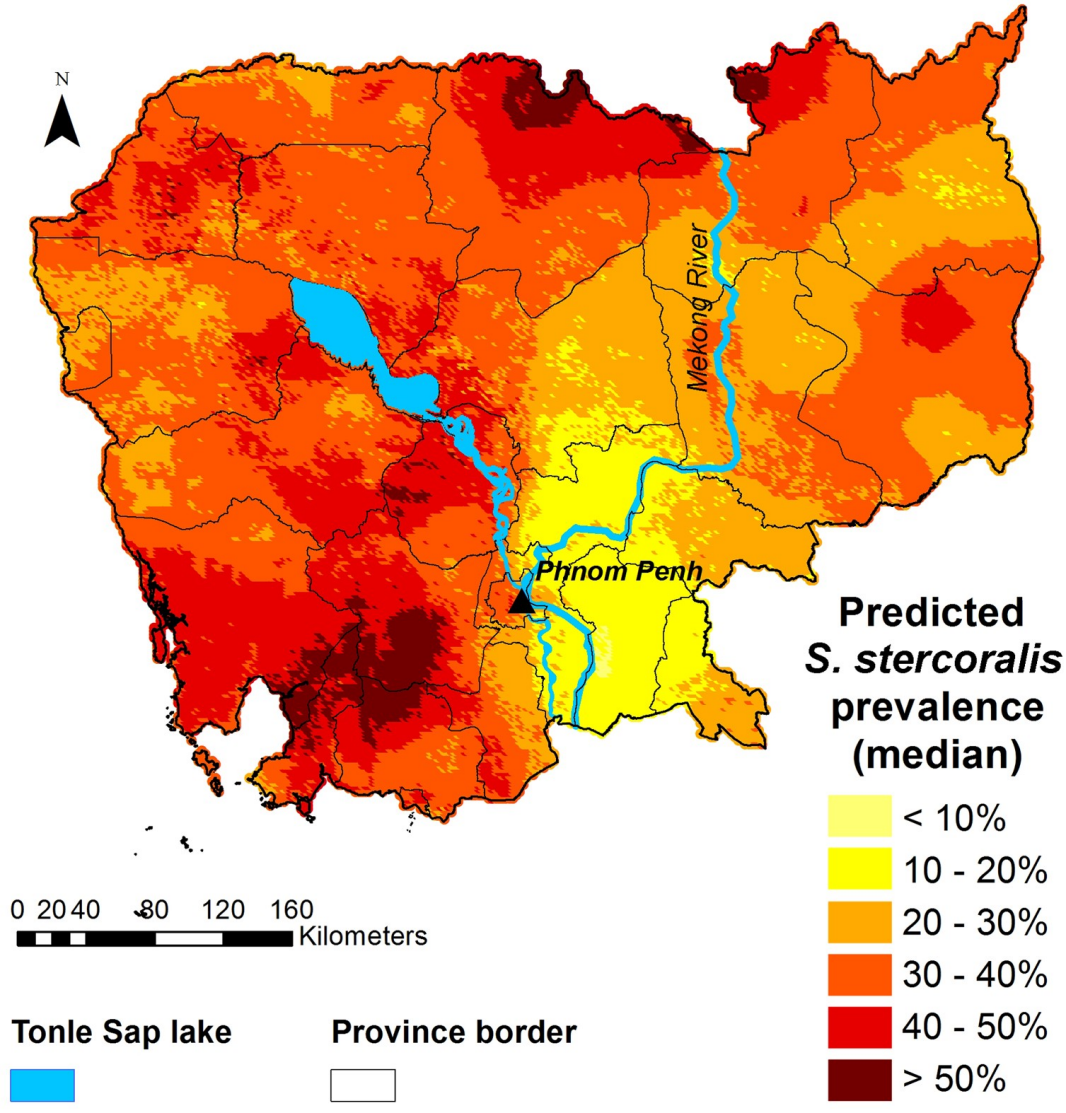
Map of the predicted prevalence (median) of *S*. *stercoralis* in Cambodia. Predictions were obtained with the geostatistical model shown in Table 3, based on survey data collected in 2016 from 7,246 participants aged 6 years and older, living in 249 villages across Cambodia. This map was created with ArcGIS version 10.0 (ESRI; Redlands, CA, USA) and display the results obtained specifically from this study by Forrer et al.

**Fig 6:**
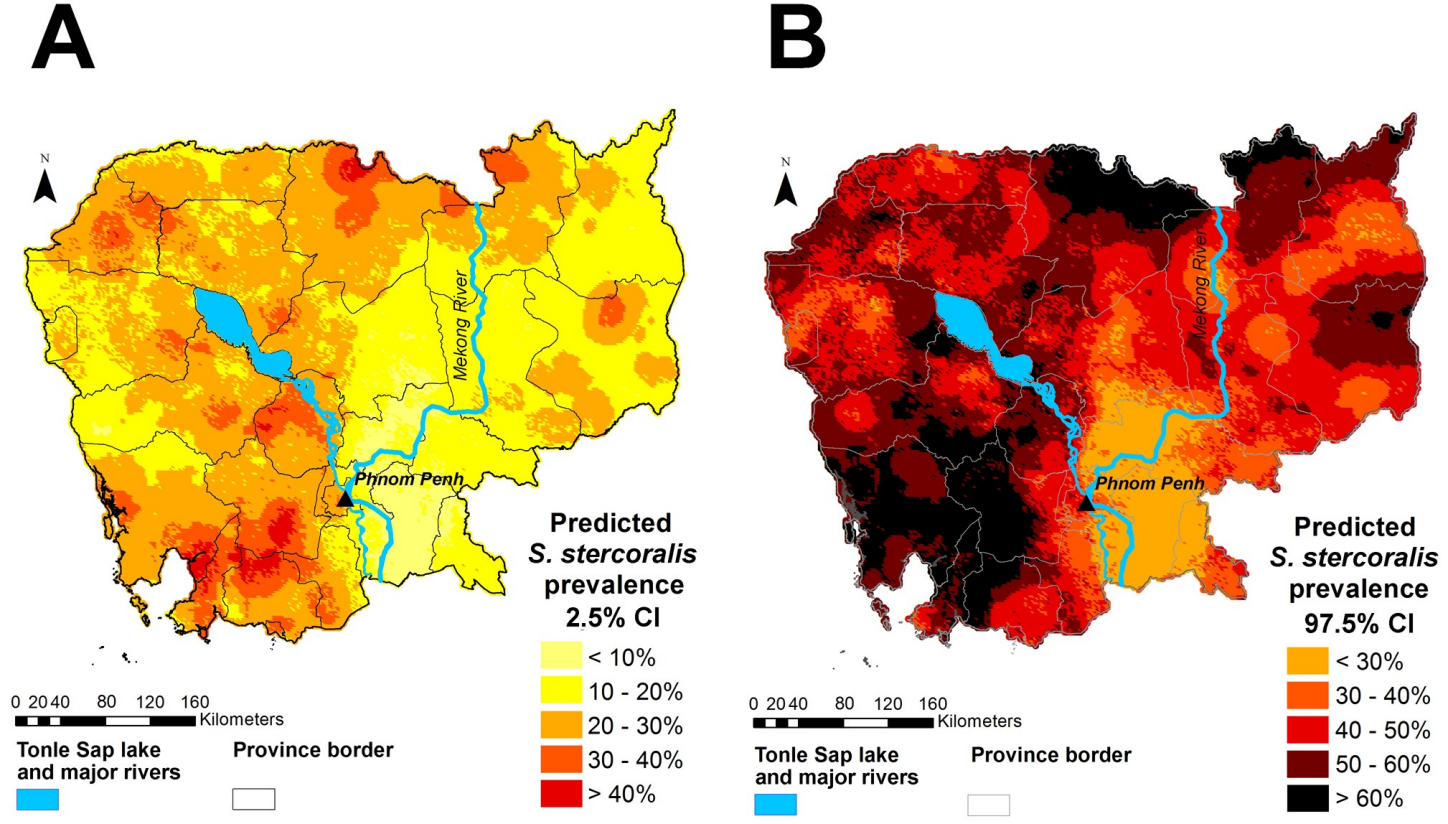
Lower (A) and upper (B) estimates of predicted *S*. *stercoralis* prevalence in Cambodia. The lower and upper estimates are the 2.5% CI and the 97.5% CI, respectively. This map was created with ArcGIS version 10.0 (ESRI; Redlands, CA, USA) and display the results obtained specifically from this study by Forrer et al.

Figs 5 and 6 display the predicted median *S*. *stercoralis* prevalence in Cambodia and the lower and upper estimates of the predictions, respectively. Prevalence was consistently higher than 10%, except in a small area of Prey Veng province. *S*. *stercoralis* predicted risk was below 20% in just five provinces, namely, Kampong Cham, Tboung Khmum, Prey Veng, Kandal and Svay Rieng. Predicted prevalence was particularly high in the north of Preah Vihear and Stung Treng provinces, near the Lao border, as well as in the south, in parts of Kampong Speu, Koh Kong, Preah Sihanouk, and Kampot provinces.

## Discussion

We present the first (to our knowledge) national prevalence estimates and nation-wide infection risk map of *S*. *stercoralis* in Cambodia, where the infection is ubiquitous. Based on a sample encompassing all 25 provinces and including more than 7,200 participants, we found that prevalence rates of *S*. *stercoralis* in Cambodia are systematically higher than 10%, with a national prevalence rate of 30%.

The risk of infection was lowest in the southeast of the country, namely the provinces of Prey Veng, Kandal, and Kampong Cham, as well as in the western and southern parts of Tboung Khmum and Kampong Thom provinces, respectively. The highest provincial-level prevalence rates, above 40%, were found in Preah Vihear in the north, Kampong Chhnang in the centre and in Koh Kong and Kampong Speu in the south.

The size of *S*. *stercoralis* infection clusters was relatively small at 85 km, similar to that observed for hookworm infection risk in the country [27]. Almost all spatial correlation of *S*. *stercoralis* infection was explained by its association with environmental factors (as indicated by the dramatic drop of the range to 3.2 km, after introducing environmental covariates into the model). This result is not surprising as, in absence of treatment, the distribution of the parasite would mostly be conditioned by its biological requirements. The predicted geographical distribution of *S*. *stercoralis* risk in this study was similar to that of hookworm prevalence among school-aged children in Cambodia, as predicted by Karagiannis-Voules and colleagues, and likely due to the two nematodes’ similar transmission routes [27]. Yet, hookworm prevalence was lower over a larger area, most likely because of the impact of ongoing STH deworming programs [27].

The odds of infection increased with increasing maximum nighttime temperature and increasing minimum rainfall. *S*. *stercoralis* larvae might have the same ability as hookworm larvae to migrate into the soil, which, in the presence of sufficient humidity, confers to the parasite a tolerance for higher temperatures [40]. The positive association between temperature and risk was more surprising, although this might relate to a particularity of *S*. *stercoralis’* life cycle. The number of females and infective larvae developing in the external environment depends on temperature, with numbers of infective larvae reaching a maximum when temperatures are 30°C and higher [11]. Hence, nighttime maximum temperatures, which range between 24°C and 32°C in Cambodia, might affect the quantity of infective larvae present in the environment.

Regarding the environmental predictors of *S*. *stercoralis* infection, neither distance to water nor the land cover category of cropland were significantly associated with infection risk in the predictive model, but they became significant in the risk factor analysis after adjusting for demographic and behavioral factors. We found a positive association between *S*. *stercoralis* infection risk and distance to water. The development and survival of *S*. *stercoralis* larvae is affected by immersion, so seasonal flooding might determine their survival in areas close to water bodies [41, 42]. Similarly, the relationship between larvae survival and water might explain the lower infection rates in areas occupied by croplands, which mostly correspond to rice fields that are regularly flooded. Yet it is also possible that distance to water captured other unmeasured features related to socio-economic factors and human activity [29]. In Cambodia, people have a clear preference for pour-flush latrines and would choose a pit latrine over a toilet, but pour-flush latrines only function with water [43]. Limited availability of water due to living farther away from permanent water bodies might result in decreased access to, or use of, sanitation facilities.

Studies that investigated risk factors for *S*. *stercoralis* infection mostly report a higher risk among men [9, 10]. This association is generally attributed to men’s extensive exposure to soil during farming activities, although the findings of our study do not support this assumption. First, in this national sample, infection risk was not associated with occupation and two-thirds of all farmers were women. Second, compared to men, only women aged 50 years and older had decreased odds of infection. The relationship between age and *S*. *stercoralis* prevalence seems to vary across settings [9, 10, 44, 45]. In this national survey of more than 7,200 individuals aged six years and older, we found that prevalence increased with age for both men and women. Previous to this national survey, in North Cambodia, prevalence was found to increase with age and reach a plateau in adulthood, while in Yunnan, China, no cases were found among individuals under the age of 15 [9, 46, 47]. Yet, no association between age and *S*. *stercoralis* infection was found in Lao PDR, South Cambodia, or Zanzibar [10, 45, 48]. Age-specific infection risk is of particular importance to target control programs and should be further documented.

Individuals who declared having some knowledge of the health problems caused by worm infections had lower odds of infection with *S*. *stercoralis*, but knowledge about the sources of infection was not associated with infection risk. While knowledge does not necessarily translate into behavior change, this result suggests that awareness of personal disease risk—which is an important driver of health promotion and increases compliance with helminth control programs—might be a better trigger of hygienic behavior than knowing exposure sources [49, 50].

The protective effect of improved sanitation against STH infection is widely acknowledged [51–55]. We found that, compared to open defecation, defecating in latrines was protective against *S*. *stercoralis* infection. This result is in line with other studies conducted in Cambodia and in Ecuador. It is also consistent with a recent meta-analysis that included nine studies investigating the impact of sanitation on *S*. *stercoralis* infection risk, and estimated a pooled OR of 0.50 (95%CI: 0.36–0.70) [9, 10, 18, 55–57]. In North Cambodia, village-level sanitation coverage was also found to reduce re-infection risk one year after treatment [47].

The present work has several limitations. First, women were overrepresented in the sample compared to the general Cambodian population; the lower prevalence among young girls and women aged 50 years and older, compared to males, might have resulted in an underestimation of the prevalence. However, our sample was representative of the 2013 Cambodian general population in terms of age [39].

Second, it was the first time that the serological diagnostic method of detecting IgG antibodies was used for a large-scale survey. This method has proven high sensitivity for *S*. *stercoralis* detection, and it does not suffer from cross-reactivity with other STHs or food-borne trematodes [22, 23]. However, validation of the method in different settings should be carried out in order to further promote its use for estimating prevalence in other settings naïve to ivermectin treatment. In a recent study using commercial ELISA kits with different types of antigens (*S*. *ratti*, *S*. *stercoralis* and rec NIE antigen) to diagnose strongyloidiasis, concordant results between urine and serum ELISA were obtained, which suggests that urine ELISA is a reliable diagnostic method [58]. Third, prevalence estimates at village level suffer from uncertainty due to the study design and should be interpreted with caution. This uncertainty might also have affected our predicted estimates, but provincial-level prevalence rates appeared to be fairly reliable and the overall sample size was reasonably large. Fourth, eight of 249 villages (7.2%) needed to be replaced after the initial selection due to their remoteness. There was reason to believe that the data from these places might be inadequate in terms of quality. Although, *S*. *stercoralis* is generally more prevalent in highly remote areas, the number of replacements were low and the geospatial modeling allowed us to predict the infection rates in these remote locations. Finally, our risk factor analysis did not adjust for socio-economic status. Although socio-economic status was found to be associated with infection risk in North Cambodia, results from the few studies that accounted for it are heterogeneous [9, 10, 47, 59, 60]. It is worth noting that socioeconomic status was not a confounder of the relationship between age or sex and *S*. *stercoralis* infection risk in North Cambodia and would probably not have substantially affected the estimates for sex and age in the present study [47, 60]. Given the strong association between poverty and other STH infections, it is likely that *S*. *stercoralis* risk distribution is also associated with socioeconomic status and future studies should account for it.

Our study represents a clear risk map of *S*. *stercoralis* in a highly endemic setting. Based on these data, the number of infected can be quantified, which allows for realistic and concrete planning of control measures. Further developing this operational approach in other settings and with other validated diagnostic approaches will result in databases for global planning. The mainstay of the WHO’s strategy to control STH is preventive chemotherapy, i.e. regular treatment of entire populations or at-risk groups with mebendazole or albendazole to prevent high intensity infections and associated morbidity [61, 62]. However, a single oral dose of either of those drugs is not efficacious against *S*. *stercoralis*, for which the drug of choice is ivermectin [63–65]. A single oral dose (200μg/kg Body Weight) of ivermectin was found to achieve a high cure rate and result in re-infection rates below 15%, one year after treatment, in a highly endemic setting in Cambodia [47, 63, 64]. As our results demonstrate, *S*. *stercoralis* is highly endemic throughout Cambodia and the inclusion of ivermectin in the control program would be required [13, 65, 66]. Yet, this drug is not subsidized in regions where onchocerciasis is absent, let alone to treat *S*. *stercoralis*. The high cost of ivermectin in Cambodia, at USD 10 per tablet (up to five tablets may be needed to treat an individual, depending on their weight) precludes the deployment of adequate control measures in the country.

In the absence of data on age-specific morbidity, the fact that individuals of any age appear to have the same risk for re-infection one year after treatment suggests a need for community-wide control [47]. Yet, a study investigating *S*. *stercoralis*-related morbidity in Cambodia found that children and adolescents with higher parasite loads had higher odds of being stunted, while *S*. *stercoralis* infection was found to be associated with anemia but not stunting in Argentina [17, 67]. The relationship between *S*. *stercoralis* parasite loads, morbidity, and transmission intensity needs to be assessed, along with age-related infection levels, using appropriately designed longitudinal studies. Cost-effectiveness studies of various control options are needed. Mathematical models could help better appraise the parasite transmission dynamics and guide control efforts, as the complex life cycle of *S*. *stercoralis* might yield transmission dynamics that differ from other STHs.

Cambodia benefits from a well-established STH control network and was among the first countries to reach the 75% national coverage target [68, 69]. STH deworming activities were recently scaled up to reach children in middle and high schools, including private schools, and women of child-bearing age, working in factories [70]. Additionally, schistosomiasis has been successfully controlled, with no severe cases recorded recently, while lymphatic filariasis has been eliminated as a public health problem and is now under surveillance for elimination [69, 71–73].

In conclusion, *S*. *stercoralis* is highly prevalent and ubiquitous in Cambodia and urgently requires control. Although Cambodia benefits from a national helminth control program that has demonstrated its capacity to efficiently address helminthic infections, the current high cost of ivermectin cannot be entirely supported by the Ministry of Health, which precludes its use for large-scale control measures. Subsidies, donations, or the production of affordable generics are necessary to start tackling this potentially dangerous parasite that infects almost a third of the Cambodian population.

## Supporting information

S1 Fig Environmental predictors. (TIF)

S1 Table Results of the bivariate non-spatial regression for individual-level risk factors. (PDF)

S1 Appendix Questionnaire, English version. (PDF)

S2 Appendix Bayesian model formulation. (PDF)

S1 Checklist Strobe statement–checklist of items that should be included in reports of cross-sectional studies. (DOC)
