# Supplementary figures and images for: Strongyloides stercoralis: Spatial distribution of a highly prevalent and ubiquitous soil-transmitted helminth in Cambodia
Source: PLoS Negl Trop Dis. 2019 Jun 20;13(6):e0006943. doi: 10.1371/journal.pntd.0006943 (PMC6586258; doi:10.1371/journal.pntd.0006943)

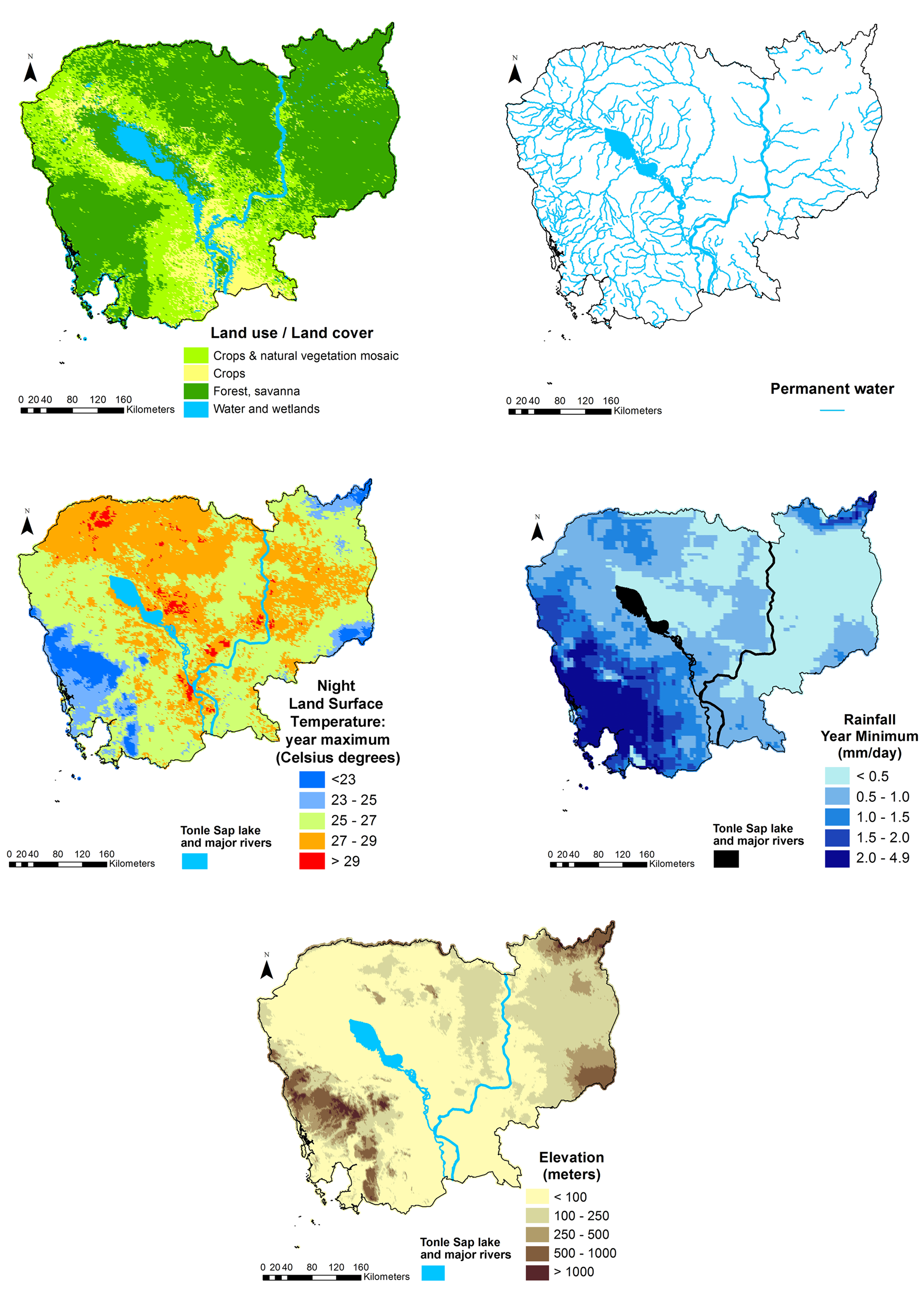

Supplement: S1 Fig — (TIF) [file pntd.0006943.s001.tif]
