# Supplementary material for: Strongyloides stercoralis: Spatial distribution of a highly prevalent and ubiquitous soil-transmitted helminth in Cambodia
Source: PLoS Negl Trop Dis. 2019 Jun 20;13(6):e0006943. doi: 10.1371/journal.pntd.0006943 (PMC6586258; doi:10.1371/journal.pntd.0006943)
Supplement: S1 Table — (PDF) [file pntd.0006943.s002.pdf]

S2 Table. Results of the bivariate non-spatial regressions for individual-level risk factors

**Variable**	**Category**	**OR**	**95% CI**	**LRT p-value**
Sex	Male	1.00	-	0.8695
	Female	1.01	0.9 -1.12	
Age group (years)	6 - 12	1.00	-	< 0.0001
	13 - 18	1.8	1.47-2.21	
	19 - 30	2.29	1.89-2.77	
	31 - 50	3.38	2.86-3.99	
	≥ 50	4.8	4.03-5.72	
Usual defecation place	Toilet	1.00	-	0.0287
	Forest	1.15	1.00-1.33	
	River, rice field, other	1.29	1.03-1.62	
Education attainment	Primary	1.00	-	< 0.0001
	No school	1.81	1.57-2.09	
	Secondary	1.17	1.01-1.35	
	High school and higher	0.97	0.78-1.21	
Main occupation	Farmer	1.00	-	< 0.0001
	At school	0.38	0.34-0.43	
	At home	0.76	0.58-0.99	
	Other	0.8	0.65-0.99	
Number of household members	-	0.99	0.96-1.01	0.2517
Do you know anything about worms?	No	1.00	-	
	Yes	1.29	1.14-1.46	< 0.0001
Do you know how to get infected with worms?	No	1.00	-	
	Yes	1.13	1.00-1.27	0.0491
Do you know what health problems worm infection can cause?	No	1.00	-	
	Yes	1.11	0.98-1.25	0.1043
Source of infection: walking barefoot [a]	No	1.00	-	
	Yes	0.97	0.83-1.13	0.6909
Source of infection: lack of hygiene [a]	No	1.00	-	
	Yes	0.99	0.87-1.13	0.8795
Source of infection: open defecation [a]	No	1.00	-	
	Yes	1.06	0.91-1.24	0.4313
	No	1.00	-	
Source of infection: no handwashing [a]	Yes	1.04	0.90-1.20	0.6236

[a] spontaneous specific answers to the question "Do you know how to get infected with worms"?

Data were obtained from a cross-sectional survey onducted in 2016 among 7,246 participants living in 249 villages across the 25 provinces of Cambodia
