# Supplementary material for: Strongyloides stercoralis: Spatial distribution of a highly prevalent and ubiquitous soil-transmitted helminth in Cambodia
Source: PLoS Negl Trop Dis. 2019 Jun 20;13(6):e0006943. doi: 10.1371/journal.pntd.0006943 (PMC6586258; doi:10.1371/journal.pntd.0006943)
Supplement: S1 Appendix — (PDF) [file pntd.0006943.s003.pdf]

Nation-wide Mapping of Instestinal Helminth Infection in Cambodia, 2016

IDENTIFICATION

1 Date of interview: |_____|_____|.|_____|_____|. 2016 (dd/mm/yyyy)	Participant IDCODE
2 Household number: |___|___| 3- Village: ____________ 4- Commune: _____________	
5 District: __________ 6- Province ____________	00-00-00<br>PRO-VIL-PP
7 Respondent's Name ________________________________	

DEMOGRAPHICS

8 Age of Respondent: |_____|_____| years

9 Sex of Respondent: ☐ 1-Male ☐ 2-Female

10 Occupation of Respondent? |_____| 1-Farmer; 2-Student; 3-Fisherman; 4-Teacher; 5-Other

a If OTHER, Specify: ____

11 Education level of Respondent? |_____| 0-No School; 1-Primary School; 2-Secondary School; 3-High School; 4-University

12 How many persons live together with you in the house? |_____||_____| persons

PERSONAL DISEASE & RISK-PERCEPTION

13 Do you know anything about worms or infection with worms? ☐ 1-Yes ☐ 0-No **If NO, Skip to 16**

14 Do you know how you get infected with worms? ☐ 1-Yes ☐ 0-No **If NO, Skip to 15**

If YES, please, describe (Do not read or prompt the answer AND Multiple answer is possible)

a Unboiled Water	☐ 1-Yes	☐ 0-Not Reported	f Not washing hands	☐ 1-Yes	☐ 0-Not Reported
b Swimming	☐ 1-Yes	☐ 0-Not Reported	g Undercooked fish	☐ 1-Yes	☐ 0-Not Reported
c Bare foot	☐ 1-Yes	☐ 0-Not Reported	h Undercooked meat	☐ 1-Yes	☐ 0-Not Reported
d Lack of hygiene	☐ 1-Yes	☐ 0-Not Reported	i Undercooked vegetables	☐ 1-Yes	☐ 0-Not Reported
e Defecate outside toilet	☐ 1-Yes	☐ 0-Not Reported	j Other, Specify: ________________________________		

15 Do you know what health problems can occur because of a worm infection? ☐ 1-Yes ☐ 0-No **If NO, Skip to 16**

If YES, please, describe (Do not read or prompt the answer AND Multiple answer is possible)

a Skin problems	☐ 1-Yes	☐ 0-Not Reported	e Vomit blood	☐ 1-Yes	☐ 0-Not Reported
b Muscle pain	☐ 1-Yes	☐ 0-Not Reported	f Vomiting	☐ 1-Yes	☐ 0-Not Reported
c Big belly	☐ 1-Yes	☐ 0-Not Reported	g Diarrhea	☐ 1-Yes	☐ 0-Not Reported
d Stomach/abodominal pain	☐ 1-Yes	☐ 0-Not Reported	h Loss of weight	☐ 1-Yes	☐ 0-Not Reported

i Other, Specify: ________________________________

16 When (which month of this year) did you receive the anthelminthic drug (MBZ/ABZ/PZQ)? _______________

17 Do you have toilet at home? ☐ 1-Yes ☐ 0-No ☐ 9-DK **If NO or DK, Skip to 18**

a is it inside or outside the house? ☐ 1-Inside ☐ 2-Outside ☐ 9-DK

18 Where do you usually defecate? |____| 1-In Toilet; 2-Forest; 3-In River; 4-Rice Field; 5-Other

a Other, Specify: ____
