# Supplementary material for: Strongyloides stercoralis: Spatial distribution of a highly prevalent and ubiquitous soil-transmitted helminth in Cambodia
Source: PLoS Negl Trop Dis. 2019 Jun 20;13(6):e0006943. doi: 10.1371/journal.pntd.0006943 (PMC6586258; doi:10.1371/journal.pntd.0006943)
Supplement: S2 Appendix — (PDF) [file pntd.0006943.s004.pdf]

Appendix S2: Bayesian Model Formulation

For the individual level risk factor analysis we assumed the infection status Y_{ij} of an individual *i* at a village *j* followed a Bernouilli distribution: $Y_{ij} \sim Be(p_{ij})$ where p_{ij} is the probability of being infected by *S. stercoralis* for an individual *i* in a village *j*.

For the predictive model using environmental variables at village level only we assumed that at each village *j*, the number of infected Y_j followed a Binomial distribution: $Y_j \sim Bin(N_j, p_j)$ where N_j is the number of screened individuals and p_j is the probability of infection.

Spatial random effects ϕ_j, accounted for unobserved spatial processes at every village *j*. Covariates β_k and random effects ϕ_j were modelled on a log *it* scale: $\log it\left(p_{ij}\right) = \alpha + \sum_{k=1}^{n} \beta_k X_{kij} + \phi_j$, with *n* being the number of covariates. The random effect $\underline{\phi} = (\phi_1, \phi_2, \ldots, \phi_j)^T$ is assumed to follow a Normal distribution $\underline{\phi} \sim N(0, \Sigma)$, where Σ is the covariance matrix. A stationary isotropic process was assumed in the present work, with the covariance matrix $\Sigma_{ij} = \sigma^2 corr_{ij}(d_{ij}, \rho)$ and an exponential correlation function $corr_{ij}(d_{ij}, \rho) = \exp(-d_{ij}, \rho)$ where d_{ij} is the shortest distance between two locations s_i and s_j, and ρ is a measure of how spatial correlation decreases with the distance. The distance at which the spatial correlation between villages gets under 5% is equal to $3/\rho$ and is called the range.

According to Bayesian modelling specification, we chose prior distributions for all parameters to be estimated. We chose a Normal distribution with a mean of zero and a variance of 100 for the regression coefficients. An inverse gamma vague prior with mean equal to 1 and variance equal to 100 was adopted for σ^2, and a uniform prior for ρ with parameters calculated as a function of the minimum and maximum distance between sampled villages:
