# Supplementary material for: Strongyloides stercoralis: Spatial distribution of a highly prevalent and ubiquitous soil-transmitted helminth in Cambodia
Source: PLoS Negl Trop Dis. 2019 Jun 20;13(6):e0006943. doi: 10.1371/journal.pntd.0006943 (PMC6586258; doi:10.1371/journal.pntd.0006943)
Supplement: S1 Checklist — (DOC) [file pntd.0006943.s005.doc]

STROBE Statement—Checklist of items that should be included in reports of ***cross-sectional studies***

|  | Item No | Recommendation |
| --- | --- | --- |
| **Title and abstract** | 1 | (*a*) Indicate the study’s design with a commonly used term in the title or the abstract  abstract |
|  |  | (*b*) Provide in the abstract an informative and balanced summary of what was done and what was found  done |
| Introduction | | |
| Background/rationale | 2 | Explain the scientific background and rationale for the investigation being reported  §1 to 4 |
| Objectives | 3 | State specific objectives, including any prespecified hypotheses  §5 |
| Methods | | |
| Study design | 4 | Present key elements of study design early in the paper  Section “Study population and design” |
| Setting | 5 | Describe the setting, locations, and relevant dates, including periods of recruitment, exposure, follow-up, and data collection  Section “study setting”, section “Study population and design” |
| Participants | 6 | Give the eligibility criteria, and the sources and methods of selection of participants  Section “Study population and design” |
| Variables | 7 | Clearly define all outcomes, exposures, predictors, potential confounders, and effect modifiers. Give diagnostic criteria, if applicable  Sections “individual risk factor data”, “Environmental data”, “Assessment of *S. stercoralis* infection” |
| Data sources/ measurement | 8* | For each variable of interest, give sources of data and details of methods of assessment (measurement). Describe comparability of assessment methods if there is more than one group |
| Bias | 9 | Describe any efforts to address potential sources of bias  Section “Statistical analysis” |
| Study size | 10 | Explain how the study size was arrived at  Section “Study population and design” |
| Quantitative variables | 11 | Explain how quantitative variables were handled in the analyses. If applicable, describe which groupings were chosen and why  Section “Data management” |
| Statistical methods | 12 | Describe all statistical methods, including those used to control for confounding  Section “Statistical analysis” |
|  |  | (*b*) Describe any methods used to examine subgroups and interactions  Section “Statistical analysis” |
|  |  | (*c*) Explain how missing data were addressed  Observations with missing data were excluded. Missing data (mostly infection status) were not addressed with imputation methods |
|  |  | (*d*) If applicable, describe analytical methods taking account of sampling strategy  n.a. |
|  |  | (*e*) Describe any sensitivity analyses  n.a. |
| Results | | |
| Participants | 13* | Report numbers of individuals at each stage of study—eg numbers potentially eligible, examined for eligibility, confirmed eligible, included in the study, completing follow-up, and analysed  Section “Study population” |
|  |  | (b) Give reasons for non-participation at each stage  Section “Study population” |
|  |  | (c) Consider use of a flow diagram  Not done. |
| Descriptive data | 14* | 1. Give characteristics of study participants (eg demographic, clinical, social) and information on exposures and potential confounders   Section “Study population”, Table 4. |
|  |  | 1. Indicate number of participants with missing data for each variable of interest   Section “Study population” |
| Outcome data | 15* | Report numbers of outcome events or summary measures  Section “*S. stercoralis* prevalence” |
| Main results | 16 | 1. Give unadjusted estimates and, if applicable, confounder-adjusted estimates and their precision (eg, 95% confidence interval). Make clear which confounders were adjusted for and why they were included   Appendix S1Table (unadjusted estimates); Table 4 (fully adjusted estimates).  Section “Risk factors for *S. stercoralis* infection” |
|  |  | (*b*) Report category boundaries when continuous variables were categorized  Table 1, Table 4 |
|  |  | If relevant, consider translating estimates of relative risk into absolute risk for a meaningful time period  n.a. |
| Other analyses | 17 | Report other analyses done—eg analyses of subgroups and interactions, and sensitivity analyses  Table 4. Sections “Risk factors for *S. stercoralis* infection”, “Spatial correlation”, Result of the model validation and predictive model” |
| Discussion | | |
| Key results | 18 | Summarise key results with reference to study objectives  § 1, 2 |
| Limitations | 19 | Discuss limitations of the study, taking into account sources of potential bias or imprecision. Discuss both direction and magnitude of any potential bias  § 12 to 14 |
| Interpretation | 20 | Give a cautious overall interpretation of results considering objectives, limitations, multiplicity of analyses, results from similar studies, and other relevant evidence  All throughout discussion |
| Generalisability | 21 | Discuss the generalisability (external validity) of the study results  § 11 |
| Other information | | |
| Funding | 22 | Give the source of funding and the role of the funders for the present study and, if applicable, for the original study on which the present article is based  Section “Funding” |

*Give information separately for exposed and unexposed groups.

**Note:** An Explanation and Elaboration article discusses each checklist item and gives methodological background and published examples of transparent reporting. The STROBE checklist is best used in conjunction with this article (freely available on the Web sites of PLoS Medicine at http://www.plosmedicine.org/, Annals of Internal Medicine at http://www.annals.org/, and Epidemiology at http://www.epidem.com/). Information on the STROBE Initiative is available at www.strobe-statement.org.
